# Effectiveness and safety of electronically delivered prescribing feedback and decision support on antibiotic use for respiratory illness in primary care: REDUCE cluster randomised trial

**DOI:** 10.1136/bmj.l236

**Published:** 2019-02-13

**Authors:** Martin C Gulliford, A Toby Prevost, Judith Charlton, Dorota Juszczyk, Jamie Soames, Lisa McDermott, Kirin Sultana, Mark Wright, Robin Fox, Alastair D Hay, Paul Little, Michael V Moore, Lucy Yardley, Mark Ashworth

**Affiliations:** 1 School of Population Health and Environmental Sciences, King’s College London, Guy’s Campus, King’s College London, London, UK; 2 NIHR Biomedical Research Centre at Guy’s and St Thomas’ Hospitals London, London, UK; 3 School of Public Health, Imperial College London, London, UK; 4 Clinical Practice Research Datalink, Medicines and Healthcare Products Regulatory Agency, London, UK; 5 The Health Centre, Bicester, Oxfordshire, UK; 6 Centre for Academic Primary Care, Bristol Medical School, Population Health Sciences, University of Bristol, Bristol, UK; 7 Primary Care Research Group, University of Southampton, Southampton, UK; 8 Department of Psychology, University of Southampton, Southampton, UK; 9 School of Psychological Science, University of Bristol, Bristol, UK

## Abstract

**Objectives:**

To evaluate the effectiveness and safety at population scale of electronically delivered prescribing feedback and decision support interventions at reducing antibiotic prescribing for self limiting respiratory tract infections.

**Design:**

Open label, two arm, cluster randomised controlled trial.

**Setting:**

UK general practices in the Clinical Practice Research Datalink, randomised between 11 November 2015 and 9 August 2016, with final follow-up on 9 August 2017.

**Participants:**

79 general practices (582 675 patient years) randomised (1:1) to antimicrobial stewardship (AMS) intervention or usual care.

**Interventions:**

AMS intervention comprised a brief training webinar, automated monthly feedback reports of antibiotic prescribing, and electronic decision support tools to inform appropriate prescribing over 12 months. Intervention components were delivered electronically, supported by a local practice champion nominated for the trial.

**Main outcome measures:**

Primary outcome was the rate of antibiotic prescriptions for respiratory tract infections from electronic health records. Serious bacterial complications were evaluated for safety. Analysis was by Poisson regression with general practice as a random effect, adjusting for covariates. Prespecified subgroup analyses by age group were reported.

**Results:**

The trial included 41 AMS practices (323 155 patient years) and 38 usual care practices (259 520 patient years). Unadjusted and adjusted rate ratios for antibiotic prescribing were 0.89 (95% confidence interval 0.68 to 1.16) and 0.88 (0.78 to 0.99, P=0.04), respectively, with prescribing rates of 98.7 per 1000 patient years for AMS (31 907 prescriptions) and 107.6 per 1000 patient years for usual care (27 923 prescriptions). Antibiotic prescribing was reduced most in adults aged 15-84 years (adjusted rate ratio 0.84, 95% confidence interval 0.75 to 0.95), with one antibiotic prescription per year avoided for every 62 patients (95% confidence interval 40 to 200). There was no evidence of effect for children younger than 15 years (adjusted rate ratio 0.96, 95% confidence interval 0.82 to 1.12) or people aged 85 years and older (0.97, 0.79 to 1.18); there was also no evidence of an increase in serious bacterial complications (0.92, 0.74 to 1.13).

**Conclusions:**

Electronically delivered interventions, integrated into practice workflow, result in moderate reductions of antibiotic prescribing for respiratory tract infections in adults, which are likely to be of importance for public health. Antibiotic prescribing to very young or old patients requires further evaluation.

**Trial registration:**

ISRCTN95232781.

**Figure:**
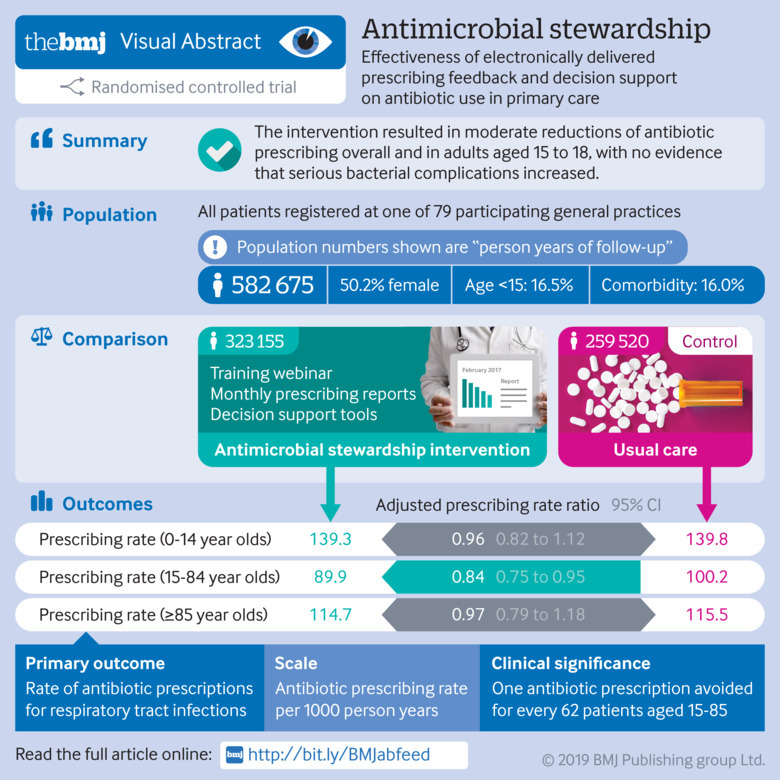


## Introduction

Overuse of antibiotics in medical practice is contributing to the emergence of antimicrobial drug resistance. The US Centers for Disease Control estimate that each year in the United States, at least two million people acquire antibiotic resistant infections and at least 23 000 people die as a direct result.^1^ General practice and ambulatory care account for nearly three quarters of all antibiotic prescribing, with respiratory tract infections (RTIs) representing the largest single group of indications for antibiotic treatment, including cough, acute bronchitis, common colds, otitis media, sinusitis, and sore throat.^2^ Antibiotic treatment generally has little if any effect on the severity or duration of RTI symptoms, is commonly associated with side effects,^3^ ^4^ and encourages patients to reconsult in future episodes.^5^ Current treatment recommendations suggest that a no antibiotic prescribing strategy should be agreed on with most patients presenting with self limiting RTIs.^6^ Only limited evidence is currently available but a strategy of reduced antibiotic prescribing does not appear to compromise patient safety in terms of bacterial infections.^7^ ^8^

A systematic review, updated to 2018, found that educational activities aimed at clinicians or patients, electronic decision support systems, and audit of antibiotic prescribing with feedback of results might be used to reduce unnecessary antibiotic prescribing.^9^ ^10^ However, the review concluded that it was unclear how useful these interventions might be in usual clinical practice because of a lack of information about possible adverse events, including possible increases in bacterial infections.^10^ Previous studies also lacked information about effect modification by patient characteristics (such as age, sex, and comorbidity) that might influence intervention effectiveness.^10^ The review suggested that a strategy of combining interventions might hold promise and recommended that trials of multifaceted interventions, including two or more interventions found to be effective individually, should be undertaken.^10^ Although some recent trials have used electronic media to deliver interventions,^11^ ^12^ previous interventions have often been resource intensive^13^ and have not yet shown potential to be translated on a wide and sustainable scale into routine healthcare. 

The present research focused on low cost interventions that can be readily integrated into routine practice workflow and scaled up through remote delivery using electronic media to a large sample of unselected practices. The research developed a multicomponent intervention that included a brief training webinar for prescribers, followed by monthly feedback reports of antibiotic prescribing at RTI consultations and decision support tools to inform appropriate prescribing. The primary objective of the present study (the REDUCE trial) was to evaluate whether this multicomponent intervention was effective and safe at reducing prescribing of antibiotics when patients consult with RTIs, when delivered electronically into general practices over 12 months.

## Methods

### Study design and participants

The study was an open label, two arm, parallel group, cluster randomised trial with general practices as the unit of allocation. The target population for this trial was the general population registered with general practices in the United Kingdom, including England, Scotland, Wales, and Northern Ireland. The trial was conducted by use of the anonymised electronic health records of general practices contributing to the UK Clinical Practice Research Datalink (CPRD). The CPRD is one of the world’s largest databases of primary care electronic health records, and it includes data updated monthly from general practices throughout the UK. CPRD data have been extensively evaluated and employed for epidemiological^14^ and interventional research.^15^ General practices contributing to CPRD were invited to participate in the study from September 2015. General practices were included in the trial if they were actively contributing data to CPRD and consented to participation in the trial. Data for all registered patients from trial practices in CPRD were included; there were no exclusion criteria.

The protocol was approved by the NHS London-Dulwich research ethics committee (14/LO/1730) and by the CPRD independent scientific advisory committee (ISAC 14_130). Trial oversight was provided by independent trial steering and data monitoring committees. Each participating general practice gave written informed consent for participation. General practices were randomised between 11 November 2015 and 9 August 2016, and final follow-up was on 9 August 2017. The trial was stopped when the last general practices completed 12 months of follow-up. The study protocol has been reported previously,^16^ and the updated protocol including amendments to the sample size calculation and statistical analysis plan has been published online.

### Randomisation and masking

Cluster randomisation was used because intervention was delivered at the general practice level. CPRD staff (JS and KS) were responsible for recruiting practices to the trial and communicating allocations but had no access to the allocation procedure. Allocation to antimicrobial stewardship and usual care trial arms was done at King’s College London (MCG, ATP) by minimisation using the MINIM program,^17^ stratifying by region and by pre-trial fourths of antibiotic prescribing (as a proportion of RTI consultations with antibiotics prescribed). “Region” comprised Scotland; Wales; Northern Ireland; and, in England, North (including North East, North West, and Yorkshire and Humber), Midlands (including East and West Midlands), South and East (including East of England, South Central, and South East Coast), South West, and London. Because only two practices were recruited in the Midlands, this region was combined with North for analysis. General practices consented to participation over an extended recruitment period; they were allocated in six waves, which were combined for analysis into three periods (period 1, practices randomised in November 2015; period 2, January and February 2016; period 3, June to August 2016). One practice allocated to the intervention trial arm withdrew from CPRD before the intervention started and was excluded from further analysis because no data were available. Health professionals were not blinded to trial arm allocation.

### Interventions

An intervention development study was conducted and is described in detail elsewhere.^16^ ^18^ Development of the antimicrobial stewardship intervention drew on social cognitive theory^19^ and self determination theory,^20^ experience from development of a previous intervention,^15^ ^21^ ^22^ and qualitative interviews with 31 prescribers to refine prototype versions of interventions. The intervention comprised three elements that were delivered remotely into practices using electronic media (table 1). A six minute, pre-recorded webinar introduced and provided brief training in use of the trial interventions. Antibiotic prescribing reports were prepared through analysis of CPRD electronic health records, which are updated monthly (supplementary figure 1). These reports were sent to each general practice by email, to present monthly updated feedback of data for counts of respiratory consultations and antibiotic prescriptions for that practice, in comparison with the preceding 12 months. Data were not analysed at the individual prescriber level because this information is not consistently available within CPRD. 

**Table 1:** Summary of final intervention content and delivery. AMR=antimicrobial drug resistance; RTI=respiratory tract infection

Intervention component and content	Delivery
**Webinar**	
Professionally produced video narrated by a practising general practitioner in a general practice setting, summarising:	Webinar delivered through electronic link embedded in trial start letter
Importance of antimicrobial drug resistance	Webinar also delivered into practice system by proprietary software with active alerting
Introduction to decision support tools	General practitioners encouraged to present and discuss webinar in practice meetings
Introduction to antibiotic prescribing reports, including reduced antibiotic prescribing and patient safety, and reduced antibiotic prescribing and patient satisfaction	—
**Antibiotic prescribing reports**	
Monthly updated reports on antibiotic prescribing for RTI, including:	Delivered by email to the general practitioner identified as champion for the trial at the practice
Professionally designed template	Requested to circulate prescribing reports to all prescribers at the practice
Data for number of RTI consultations and antibiotic prescriptions for RTI aggregated by month	General practitioners encouraged to discuss prescribing reports in practice meetings
Automated calculation of estimates written into a template using a software program written in R	Provided evidence of audit for professional appraisals
Data presented as table and barchart in PDF document	—
Comparison with previous year at the same practice	—
Accompanying commentary and links to decision support tools	—
**Decision support tools**	
Professionally designed decision support tools, including:	Delivered into general practice systems through proprietary software
Printable patient information leaflets for cough and bronchitis, otitis media, sinusitis, sore throat, and common cold and upper respiratory tract infection	Activated during consultations when medical codes for RTI were entered into patient electronic records
Versions for children with otitis media, and cough and bronchitis	—
Advice to patients and carers on expected duration of illness, expected course and lack of effect of antibiotics, recommendations for self care, and advice on appropriate reasons to seek help	—
Summary for prescribers of the indications for which an antibiotic prescription is usually necessary, based on national recommendations	—

Decision support tools were deployed remotely into existing practice software to provide patient information sheets and advice on the positive indications for antibiotic prescription during consultations for RTI (supplementary figure 2). Patient information sheets were provided for common colds and upper respiratory infections, sore throat, otitis media, sinusitis, and cough and bronchitis (supplementary figure 3). Separate sheets for children were provided for otitis media and cough and bronchitis. Recommendations for positive indications for antibiotic prescription followed NICE guidance (supplementary figure 4).^6^ Intervention materials were accessible to all prescribers in antimicrobial stewardship trial arm practices. General practices were asked to identify a general practitioner as a champion for the study, generally the research coordinator at the practice, who ensured that all prescribers at the practice were aware of the trial, learned how to use the decision support tools, and received copies of the antibiotic prescribing feedback reports each month. Practices were encouraged to discuss the webinar and antibiotic prescribing feedback reports at practice meetings. A more extensive description of the intervention is published elsewhere.^18^ There were no modifications during the course of the study. General practices randomised to usual care received no study interventions.

### Outcomes

Outcomes for both trial arms of antimicrobial stewardship and usual care were evaluated by use of anonymised electronic health records of individual patients from CPRD. Consultations for self limiting RTIs were identified from medical codes for cough and bronchitis, otitis media, rhinosinusitis, sore throat, and common colds. Medical codes were drawn from the Read code classification (supplementary tables 6-7), which was in use in the UK at this time, including symptoms and medical diagnoses. Lower respiratory tract infections including “chest infections,” exacerbations of chronic bronchitis, and pneumonia were not included because they are subject to different treatment recommendations. Antibiotic prescriptions were identified from product codes for antibiotics included in the *British National Formulary* section 5.1. We determined whether antibiotics were prescribed on the same date as the RTI consultation. In order to focus on prescribing decisions at initial presentations for RTI, we excluded repeat consultations for RTI during the same episode using a 14 day time window. As sensitivity analyses, we evaluated whether estimates differed for a 10 day window or no time window. 

The primary outcome measure was the rate of antibiotic prescribing for RTI per 1000 patient years over the 12 month intervention period. Secondary outcome measures were the proportion of RTI consultations with antibiotics prescribed, the consultation rate for RTI per 1000 patient years, antibiotic prescribing for subgroups of RTI, and total antibiotic prescribing for all indications. Safety outcomes were identified by the data monitoring committee as new occurrences of a wide range of serious bacterial complications including pneumonia, peritonsillar abscess, mastoiditis, intracranial abscess, empyema, scarlet fever, pyelonephritis, septic arthritis, osteomyelitis, meningitis, toxic shock syndrome and septicaemia, and Lemierre’s syndrome. Interim analyses of safety outcomes were presented to the data monitoring committee in October 2016 and April 2017. The comorbidity status of patients consulting with RTI was classified as present or absent based on “seasonal flu at-risk” status including diagnoses of significant heart, lung, renal, liver, or neuromuscular disease, as well as cystic fibrosis, diabetes, and immunosuppression or immunosuppressive treatment.^23^

### Statistical analysis

The primary outcome for the trial was the rate of antibiotic prescriptions for RTI per 1000 patient years. The sample size calculation was based on analysis of outcomes aggregated to cluster level; it was informed by data from our previous trial^15^ in CPRD. The distribution of general practices’ prescribing rates of antibiotics had a mean 111.9 (standard deviation 39.8) prescriptions per 1000 patient years, with a correlation coefficient of 0.82 between prescribing in the baseline and intervention periods. We initially aimed to recruit 120 CPRD general practices. Based on analysis of covariance, this number would have enabled the study to detect an absolute reduction in antibiotic prescribing for RTI of 12 per 1000 registered patients. During the recruitment phase of the trial, this target was not achieved because of declining numbers of general practices using “Vision” software contributing to CPRD. An updated sample size calculation on 11 July 2016 found that that a revised total of 80 practices would give 80% power (with α=0.05) to detect an absolute reduction in antibiotic prescribing rate of 15 per 1000 patient years.

Individual level patient data for primary, secondary, and safety outcomes were analysed according to the intention-to-treat principle. The original protocol^16^ proposed a general practice-level analysis, but this was amended in the statistical analysis plan, and approved by the CPRD independent scientific advisory committee, because attrition of practices during the trial and increased focus on safety outcomes^7^ required consideration of covariates at the level of individual patients, as well as consideration of temporal effects in an individual level analysis.

The trial dataset comprised full electronic health records data for all patients who consulted with RTI on one or more occasions during the trial baseline and intervention periods, together with denominator data for all patients registered at trial practices. For each registered patient, we evaluated the person time at risk during the 12 month intervention period of the trial. A random effects Poisson model was fitted using the “hglm” package in the R program,^24^ with a random intercept for general practice and the log of person years as offset. The dependent variable was a count of antibiotic prescriptions. Covariates were trial arm, sex, age group, comorbidity status, region, study quarter, and baseline prescribing rate of antibiotics. The period of randomisation was included, as well as the interaction of period with the baseline prescribing rate. The baseline prescribing rate of antibiotics was included as an age standardised rate for each practice, using the European standard population for reference. For practices that withdrew during the intervention period, baseline time was included pro rata. Forest plots were constructed. We did a sensitivity analysis for the primary outcome by fitting an overdispersed Poisson model using the “dhglm” package in the R program.^24^

Data were visualised by calculating antibiotic prescribing rates for RTI by a single year of age and fitting smoothed curves using third degree polynomials. These empirical data were compared with estimates from a fully adjusted random effects Poisson model incorporating a third order polynomial term for age and the interaction with trial arm, with age 15 years used as reference.

Safety outcomes were ascertained from CPRD clinical and referral files. The referral files include coded data for hospital referrals and discharge letters. We analysed safety outcomes adjusting for age group, sex, and comorbidity. A random effect for general practice was included for the most common outcome of pneumonia and for the composite, but this was omitted for the remaining outcomes.

Interaction terms were tested and prespecified subgroup analyses were conducted. The statistical analysis plan included prespecified subgroup analyses by age group, sex, comorbidity, region, type of infection, and baseline antibiotic prescribing fourth. Age group was categorised from 0 to 14 years, then in 10 year bands, until 85 years and over. The subgroup effect was assessed statistically on this basis and the effect was summarised more simply in those aged 0-14 years classed as children, those aged 15-84 years, and those aged 85 years and over.

Data on use of decision support tools was collected directly into the proprietary software used to deliver the tools. For each general practice, we estimated the proportion (%) of RTI consultations at which decision support tools were viewed, and we evaluated a linear trend for the primary outcome across fourths of decision support tool use adjusting for the same covariates.

### Patient and public involvement

The trial procedure and proposed intervention were presented to a participation group of patients in primary care, and feedback and views were obtained on all aspects of the intervention including the way in which messages would appear on general practitioners’ screens, and information that would be presented to patients.

## Results

**Fig 1:**
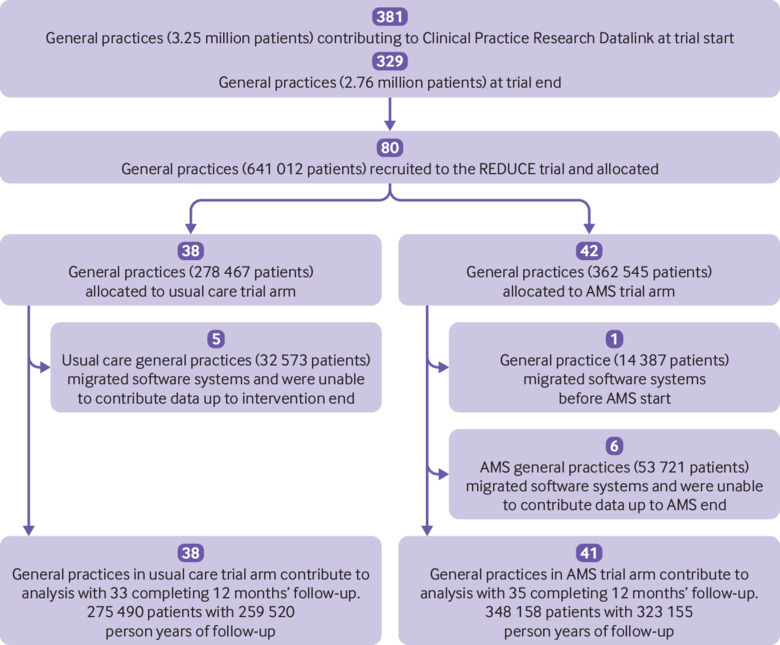
Flowchart showing trial general practices and registered populations. Numbers of patients are those registered with practices and contributing data in the baseline period except where indicated. AMS=antimicrobial stewardship intervention

Of 80 general practices recruited to the trial, one withdrew from CPRD before the start of intervention and the remaining 79 were included in the intention-to-treat analysis (fig 1). The trial included general practices from throughout the UK (table 2), and the registered population included patients of all ages. RTI consultation and antibiotic prescribing rates were similar overall between trial arms but showed wide variation among practices (table 2). General practices at in the antimicrobial stewardship arm had slightly higher numbers of registered patients than the usual care arm, but the range of practice sizes was similar across both trial arms.

**Table 2:** Characteristics of trial general practices and patient populations at baseline

	Trial arm	
	Antimicrobial stewardship	Usual care
**General practices**		
Number	41	38
Region		
London	4 (9.8)	3 (7.9)
Midlands and North England	4 (9.8)	4 (10.5)
Northern Ireland	4 (9.8)	5 (13.2)
Scotland	10 (24.4)	9 (23.7)
South and East England	8 (19.5)	6 (15.8)
South West England	3 (7.3)	4 (10.5)
Wales	8 (19.5)	7 (18.4)
Period of randomisation		
November 2015	7 (17.1)	11 (28.9)
January-February 2016	18 (43.9)	13 (34.2)
June-August 2016	16 (39.0)	14 (36.8)
Practice list size (median (range))	8936 (1086-18 425)	6777 (2530-18 557)
**Total registered patients**		
Number	348 158	278 467
Age group (years)		
<15	55 577 (16.0)	47 509 (17.1)
15-24	40 544 (11.6)	30 610 (11.0)
25-34	45 545 (13.1)	37 444 (13.4)
35-44	46 288 (13.3)	38 766 (13.9)
45-54	52 447 (15.1)	41 507 (14.9)
55-64	42 275 (12.1)	33 769 (12.1)
65-74	35 746 (10.3)	26 760 (9.6)
75-84	20 919 (6.0)	15 264 (5.5)
≥85	8817 (2.5)	6838 (2.5)
Sex		
** **Male	173 383 (49.8)	138 588 (49.8)
Female	174 775 (50.2)	139 879 (50.2)
Comorbidity		
No	288 594 (82.9)	238 106 (85.5)
Yes	59 564 (17.1)	40 361 (14.5)
Antibiotic prescribing rate (No per 1000 patient years; median (range))*	108 (4-244)	114 (20-266)
RTI consultation rate (No per 1000 patient years; median (range))*	261 (11-454)	261 (76-526)
Proportion of RTI consultations with antibiotics prescribed (%; median (range))*	43 (12-64)	43 (24-78)

Data are number (% of column total) unless indicated otherwise. RTI=respiratory tract infection.

* Numbers were age standardised, using the European standard population for reference.

### Primary outcome

**Fig 2:**
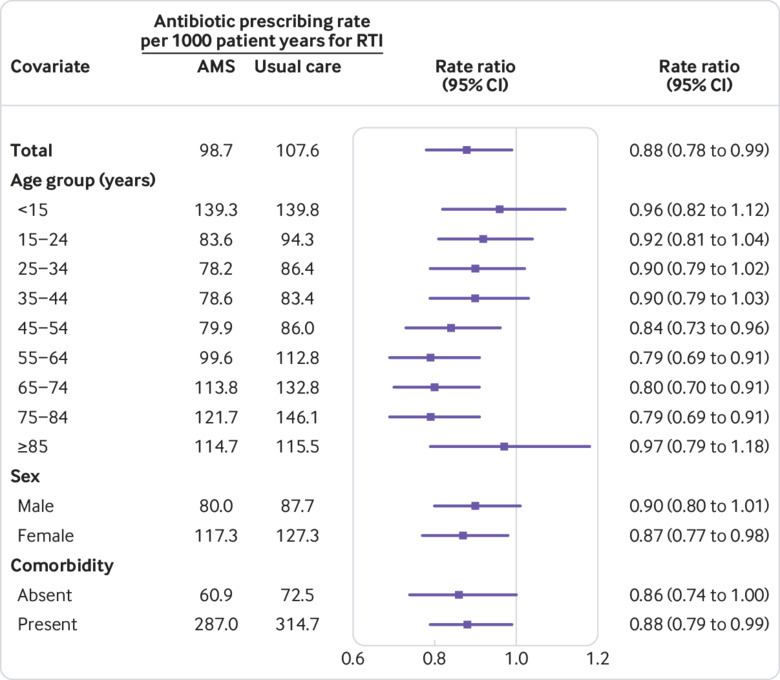
Effect of antimicrobial stewardship or usual care on primary outcome of antibiotic prescribing rate for self limiting respiratory tract infection. Estimates adjusted for random effect of general practice and covariates including sex, age group, comorbidity, region, quarter in study, practice specific rate at baseline, and interaction with period of randomisation. AMS=antimicrobial stewardship intervention; RTI=respiratory tract infections

Figure 2 presents the results for analysis of the primary outcome. The adjusted rate ratio for antibiotic prescribing for RTI was 0.88 (95% confidence interval 0.78 to 0.99, P=0.04). The antimicrobial stewardship trial arm had 31 907 antibiotic prescriptions for RTI during 323 155 patient years at 41 practices, with 98.7 prescriptions per 1000 patient years. The usual care trial arm had 27 923 prescriptions during 259 520 person years at 38 practices, with 107.6 prescriptions per 1000 patient years. Adjustment for covariates was pre-planned, before analysis, in order to improve the precision of estimated intervention effects. For comparison, the unadjusted rate ratio would have been 0.89 (0.68 to 1.16). In an analysis of data aggregated to general practice level, the mean difference in the age standardised rate of antibiotic prescribing was −0.5 (95% confidence interval −8.2 to 7.2) antibiotic prescriptions per 1000 patient years. 

These imprecise estimates resulted from wide variations in antibiotic prescribing between general practices; the data appeared to be overdispersed with several extreme values. The coefficient of variation of general practice specific rates of antibiotic prescribing was 0.51. Adjusting for covariates reduced the standard error of the coefficient, which was largely accounted for by adjustment for practices’ antibiotic prescribing for RTI at baseline. In a sensitivity analysis, an overdispersed Poisson model gave an adjusted rate ratio of 0.86 (95% confidence interval 0.75 to 0.97), which confirmed conclusions. Sensitivity analyses, which compared the base case 14 day time window (for excluding secondary consultations) with either a 10 day time window or no time window, showed that the effect estimate was not sensitive to whether a time window was used or its length (supplementary table 1).

### Secondary outcomes

We saw insufficient evidence for a difference between trial arms for the consultation rate for self limiting respiratory infections (rate ratio 0.94, 95% confidence interval 0.86 to 1.03), proportion of consultations with antibiotics prescribed (where RTI consultations rather than person time represented the denominator; 0.96, 0.89 to 1.03), and antibiotic prescribing for all indications (0.93, 0.83 to 1.04; supplementary table 2).^16^ During the intervention period, 185 924 antibiotic prescriptions were made in the AMS trial arm and 150 539 prescriptions in the usual care trial arm (supplementary table 2).

### Safety outcomes

**Fig 3:**
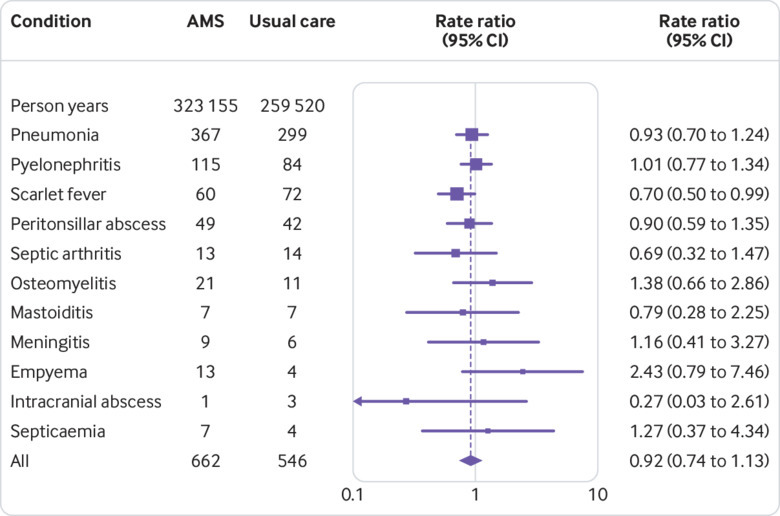
Forest plot showing rate ratios (95% confidence interval) of safety outcomes in antimicrobial stewardship trial arm compared with usual care trial arm as reference. Data are frequencies except where indicated. Estimates were from a Poisson model adjusted for age group, sex, and comorbidity. Analyses for pneumonia and combined outcome were adjusted for random effect of general practice. One case of Lemierre’s syndrome in the usual care arm not shown. AMS=antimicrobial stewardship intervention

Figure 3 presents numbers of serious bacterial complications by trial arm, together with a forest plot of rate ratios. We saw no evidence to suggest that bacterial infections were more frequent in the antimicrobial stewardship arm (rate ratio 0.92, 95% confidence interval 0.74 to 1.13) than in the usual care arm. The usual care arm had slightly more events of scarlet fever, and the antimicrobial stewardship arm had slightly more empyema events, but these were likely to be chance findings. The usual care arm had one case of Lemierre’s syndrome. We saw no evidence that the adjusted rate ratio varied by age group (χ^2^=1.228, df=8, P=0.99); the adjusted rate ratio was 0.82 (0.44 to 1.51) for children and 0.99 (0.59 to 1.70) for people aged 85 years and older.

### Subgroup analyses

Subgroup analyses by individual patient characteristics are shown in figure 2. Antibiotic prescribing was strongly associated with age (Wald test of the trial arm by age group interaction, χ^2^=65.5, df=8, P<0.001). Results of a prespecified subgroup analysis by age are shown in figure 2. We saw no evidence of an effect of intervention in children aged under 15 years (rate ratio 0.96, 95% confidence interval 0.82 to 1.12) or in people aged 85 years or older (0.97, 0.79 to 1.18). In the usual care trial arm, children accounted for 6432 (23%) antibiotic prescriptions, while people aged 85 years and older accounted for 680 (2%). At intermediate ages, antibiotic prescribing was lower in the antimicrobial stewardship arm than in the usual care arm. We summarised effect modification by age by comparing effect measures in children, adults aged 15-84 years, and people aged 85 years and older (supplementary table 3). The intervention was associated with lower antibiotic prescribing for RTI in adults aged 15-84 years (0.84, 0.75 to 0.95). Based on the antibiotic prescribing rate for adults aged 15-84 years in the usual care arm of 100.2 per 1000 patient years, the absolute risk reduction was 16.0 (5.0 to 25.1) antibiotic prescriptions per 1000 patient years. This is equivalent to the saving of one antibiotic prescription per year for every 62 registered patients (95% confidence interval 40 to 200) aged 15-84 years.

**Fig 4:**
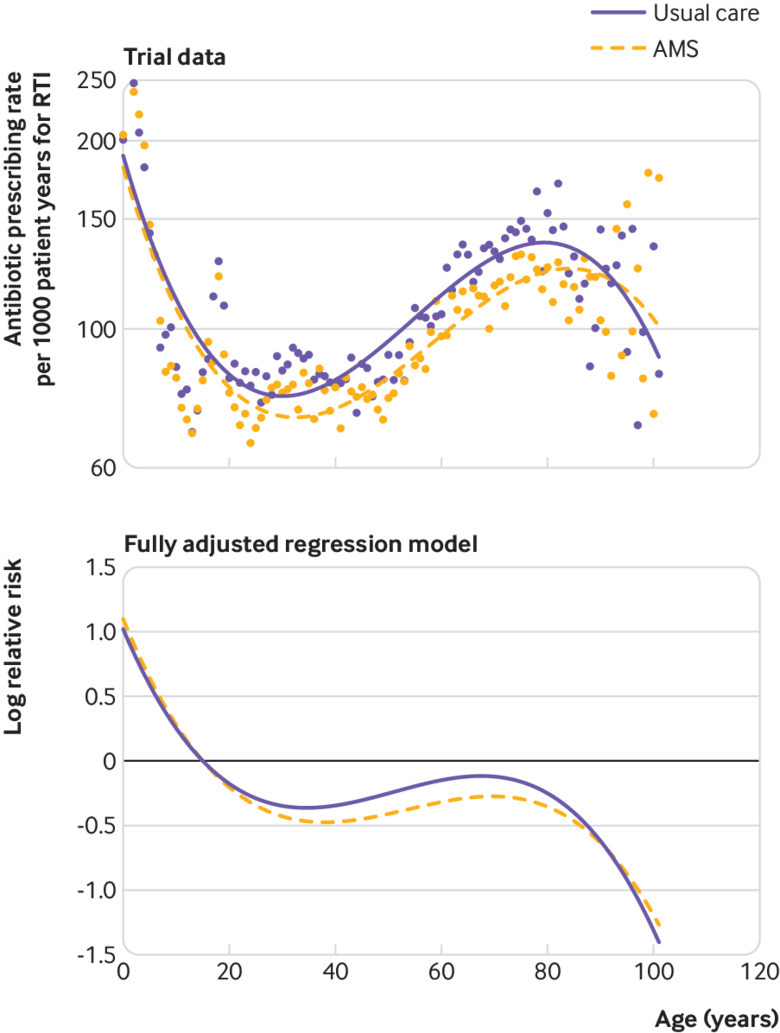
Comparison of antibiotic prescribing by single year of age for antimicrobial stewardship and usual care trial arms. Top panel: antibiotic prescribing rates per 1000 patient years by single year of age, with fitted third order polynomial curve. The y axis uses a log scale. Bottom panel: log relative risk estimates from random effects Poisson model using age 15 years for reference; log relative risk estimates were adjusted for random effect of general practice and covariates including sex, age group, comorbidity, region, quarter in study, practice specific rate at baseline, and interaction with period of randomisation. AMS=antimicrobial stewardship intervention; RTI=respiratory tract infections

Figure 4 presents empirical trial data for antibiotic prescribing rates for RTI by single year of age. Fitted polynomial curves suggest that from the late teens to the early eighties, antibiotic prescribing for RTI was lower in the intervention trial arm, but there was no evidence of reduced antibiotic prescribing in children or very old people. Estimates from the fully adjusted regression model (fig 4) show the same pattern of effect with evidence of reduced prescribing in the intervention trial arm from the late teens to early eighties. Data were relatively sparse at very advanced ages, with fewer than 500 patient years’ follow-up at any single year of age over 90 years.

There was no evidence that the effect of intervention might differ by sex (χ^2^=1.264, df=1, P=0.26) or comorbidity (χ^2^=2.424, df=1, P=0.12). Analysis by subgroup of practice level covariates (region and baseline antibiotic prescribing fourth) showed no consistent pattern of association (supplementary table 4). We saw no evidence of association of intervention with antibiotic prescribing for any subgroup of RTI separately (supplementary table 5).

### Process evaluation

We evaluated the primary outcome measure in relation to the level of use of decision support tools at practices in the antimicrobial stewardship trial arm. In the lowest fourth of use, decision support tools were viewed at fewer than 1% of RTI consultations but up to 28% in the fourth with the highest use (table 3). We saw evidence of a linear trend in the primary outcome across fourths of decision support tool use, with relative risk reduction of 3.4% per fourth (95% confidence interval 0.1% to 6.5%). This association appeared to be stronger for adults, with no evidence of association for children or people aged 85 or older (table 3). In the intervention period, the number of patient information leaflets printed per practice ranged from zero to 555, with median 54 (interquartile range 7-97) leaflets printed per practice.

**Table 3:** Association of antibiotic prescribing rate for self limiting respiratory tract infection with use of decision support tools, by age group

Fourth of decision support tool use	RTI consultations with DST viewed (%)	Antibiotic prescribing for respiratory tract infection/No of person years)			
		All	Age 0-14 years	Age 15-84 years	Age ≥85 years
Usual care trial arm	—	27 923/259 519.7	6432/46 019.6	20 811/207 611.4	680/5888.7
Lowest fourth	0 to 0.6	7190/85 805.1	1932/15 699.9	5089/68 220.1	169/1885.1
Second fourth	0.6 to 2.9	7765/74 868.3	1706/12 009.4	5837/60 825.5	222/2033.4
Third fourth	2.9 to 6.1	10 647/91 986.9	2339/15 233.4	7957/74 735.5	351/2018.0
Highest fourth	6.1 to 27.6	6305/70 495.1	1520/10 883.6	4668/58 060.1	117/1551.3
Relative risk reduction (%; 95% CI) per fourth increase in decision support tools*†	—	3.4 (0.1 to 6.5)	1.60 (−2.7 to 5.7)	4.3 (1.1 to 7.5)	1.0 (−4.6 to 6.3)
P value	—	0.04	—	—	—

* Adjusted for random effect of general practice and fixed effects of sex, age group, comorbidity, region, quarter in study, practice specific rate at baseline, and interaction with period.

† Rate ratio represents the reduction in antibiotic prescribing per fourth increase in decision support tools.

## Discussions

### Principal findings of the study

In a nationwide sample of general practices, a low cost, remotely delivered intervention using electronic health records data to provide antibiotic prescribing feedback reporting, together with computer delivered decision support tools, was effective at reducing antibiotic prescribing for self limiting RTIs to adults. The reduction in antibiotic use was greater at practices that used the trial intervention (decision support tools) more frequently. We saw no evidence that the intervention was effective at reducing antibiotic prescribing to children or people aged 85 years or older. The trial decision support tools specifically addressed common diagnostic concerns in children, including otitis media and cough and bronchitis, but prescribing to the youngest and oldest age groups could be more difficult to modify because safety concerns might be more salient at these ages.^25^ Conversely, unnecessary prescribing might be more frequent, and possibly more readily modified, at intermediate ages.^26^ The intervention was delivered at low cost. The budget for the trial, including research costs, was £533 580 (€603 316; $687 802), which implies that the research and intervention were delivered for less than £1 per patient year. The marginal cost of extending the intervention to more practices might be lower. Additional analysis found no evidence that overall costs of healthcare use were different in the AMS and usual care trial arms.^18^

The trial was designed with the antibiotic prescribing rate as the primary outcome because such prescribing can influence subsequent consultation patterns for respiratory illness. Patients are more likely to consult for RTI if they have been prescribed antibiotics recently.^5^ The effect of antimicrobial stewardship interventions on prescribing could be partly mediated by changes in RTI consultation patterns. Consequently, measures such as the proportion of RTI consultations with antibiotics prescribed could underestimate intervention effects. This study did not find sufficient evidence that the proportion of RTI consultations with antibiotics prescribed or the rate of RTI consultations were reduced by the antimicrobial stewardship intervention, but both measures tended to be slightly lower in the antimicrobial stewardship arm than in the usual care arm.

The study did not find evidence that the intervention might influence the total use of antibiotics at these practices. Antibiotic prescriptions that are clearly associated with a documented RTI represented a substantial proportion of prescriptions, but nevertheless a minority consistent with another recent study,^27^ because an appreciable proportion of antibiotic prescriptions are associated with non-specific medical codes or with no code recorded. Future studies should therefore address a wider range of prescribing indications as well as issues of coding quality. We also note that only about a quarter of eligible general practices agreed to participate in the trial, and if this level of uptake were to be replicated in any future intervention rollout, then any possible population benefits would be proportionately smaller.^28^ The trial continued over 12 months and it did not provide evidence concerning any possible longer term outcomes. The trial intervention did not address selection of different antimicrobial drugs, though nationally there has been a substantial reduction in prescribing of broad spectrum antibiotics in recent years.29

Analysis of electronic health records data for serious bacterial complications as safety outcomes showed no difference between trial arms. This study was considerably larger than most previous studies, but was nevertheless not designed to provide conclusive evidence concerning the safety of reducing antibiotic prescribing. Even a much larger study might have limited power to evaluate less frequent safety outcomes or vulnerable subgroups with precision.^7^ The confidence intervals for several individual diagnoses including meningitis, empyema, and sepsis were wide because these outcomes are infrequent but nevertheless could have a high impact on affected individuals. Outcomes were ascertained from Read codes recorded in primary care electronic records. Additional information might have been obtained through linked hospital records (Hospital Episodes Statistics), but these data were not available for all trial practices. Safety outcomes were evaluated during the 12 month intervention period, but some safety events might take longer to become apparent.

### Strengths and limitations of study

The trial was conducted in the context of national efforts to reduce unnecessary antibiotic prescribing in primary care that might have affected both trial arms with possible underestimation of intervention effects. Trial general practices represented all parts of the UK, but CPRD general practices in Scotland, Wales, and Northern Ireland were more likely to agree to participate in the trial than practices in England. General practices that agreed to take part might have been more motivated to reduce antibiotic prescribing. It is known that participation in research studies might cause individuals to alter their behaviour.^30^ Prescribing feedback delivered to the intervention trial arm might have contributed to heightened awareness of research participation and could have influenced antibiotic prescribing patterns; smaller changes might be observed if prescribing feedback is used outside of the context of a research study. 

The number of practices included was smaller than originally intended and several practices were unable to continue with the trial because they transferred to a different practice information system. In our previous study, allocation was stratified by practice list size and region,^15^ but the present trial’s allocation was stratified by antibiotic prescribing fourth and region. Consequently, there was good balance between trial arms for baseline antibiotic prescribing for RTI, but trial arms were less well balanced with respect to practice list sizes. However, the range of practice list sizes was similar in both trial arms. 

We saw wide variation in antibiotic prescribing for RTI between different general practices in both trial arms, which is consistent with data that we and others have reported previously.^2^ ^31^ Consequently, the primary measure of effect was estimated imprecisely and neither a smaller effect nor a larger effect can be excluded. Based on subgroup analysis, we caution that the intervention appeared to be effective in adults but might not have an effect on prescribing to children or people aged 85 and older. We acknowledge that evaluation of multiple subgroup analyses might lead to false positive interpretations. However, our interpretation was guided by interaction tests, which provided strong evidence that the intervention effect varied by age group but not by sex or comorbidity. We do not present P values within subgroups. 

We found from analysis of data captured by the intervention delivery software that use of decision support tools was associated with effect size, which adds evidence of a causal association. Decision support tools were used at a minority of consultations but it is possible that learning from the tools might be applied in consultations in which they were not viewed. Decision support tools were necessarily triggered when prescribers entered medical codes into the practice system. Some general practitioners might record data after the end of the consultation, or might rely on free text entries, reducing the immediacy of this component of the intervention, this post-consultation exposure might contribute to the effect of the intervention over time. 

All prescribers also received antibiotic prescribing reports but we were not able to determine whether all prescribers read these each month. There is likely to have been variation among prescribers within practices, but we did not have consistent data for the prescriber level and no information concerning prescriber characteristics. We analysed data for antibiotic prescriptions issued by trial general practices. Patients might have received antibiotic prescriptions at consultations with walk-in centres and out-of-hours or emergency services, and these alternative patterns of antibiotic use might differ between trial arms. Additional data sources will be needed to evaluate this possibility. Altered diagnostic code selection might have occurred in order to justify antibiotic prescriptions,^32^ so we included both symptoms and diagnosis codes to limit this. Safety outcomes were ascertained from medical codes in primary care records and we were not aware of whether any confirmatory tests might have been performed. There was necessarily no blinding of general practice staff to the intervention.

### Comparison with other studies

Previous studies of audit and feedback interventions across a range of indications show that these often have only small effects,^33^ although some studies report larger effects.^34^ Roshanov and colleagues^33^ found that feedback interventions that provide advice to patients as well as physicians are associated with greater chance of success. This was exemplified in the REDUCE trial’s decision support tools, which offered patient information leaflets that could be viewed online or printed, as well as offering advice to physicians on the recognised indications for giving an antibiotic prescription. 

Gjelstad and colleagues^35^ reported a comparable effect from face-to-face delivery of feedback and guideline recommendations in a study from Norway. A recent trial reported on the outcome of quarterly feedback on antibiotic prescribing over two years among 2900 Swiss physicians.^36^ Over the first and second years of the trial, there was no difference in prescribing to all patients but there was evidence of reduced prescribing to adults aged 19-65 years that was not consistently observed over time. The feedback used by Hemkens and colleagues^36^ was less immediate, being provided quarterly rather than monthly. Additionally, Switzerland already has low antibiotic prescribing rates.^37^

Hallsworth and colleagues^38^ reported a reduction in antibiotic use following social norm feedback in a study focused on high prescribing general practices. A study of dental practices in Scotland found that feedback of past antibiotic prescription data was associated with a 5.7% relative reduction in antibiotic prescribing over 12 months.^39^ Audit and feedback has also been used successfully to reduce other forms of high risk prescribing in primary care.^40^ However, purposely designed interventions might be more effective for prescribing to children.^41^

### Conclusions and policy implications

In this cluster randomised trial, an antimicrobial stewardship intervention that was delivered remotely into practices and integrated into routine care delivery was associated with a 12% reduction in antibiotic prescriptions for RTI overall, with no evidence of increased serious bacterial complications. Although the absolute impact is moderate, it is likely to be important for public health in the drive to reduce antibiotic prescribing and the risks of antimicrobial resistance. We caution that the intervention might not be effective at reducing antibiotic use for children or people aged 85 or older. Interventions using data from electronic health records might be used to promote antimicrobial stewardship in primary care and might be readily scaled up. The needs of very young or old patients need specific consideration. Our trial results also suggest the need for further research into the safety of reduced prescribing.

**What is already known on this topic**

Widespread unnecessary prescribing of antibiotics is contributing to the emergence of antimicrobial drug resistance
A systematic review of antimicrobial stewardship interventions suggested that single interventions including patient and public education, point-of-care testing, audit and feedback, and electronic decision support might be associated with reduced antibiotic use
The relevance of previous trials to clinical practice is also unclear because of limited reporting of adverse clinical outcomes and lack of detail concerning possible effect modifiers, including patient characteristics

**What this study adds**

This large UK study used data from electronic health records (EHRs) to evaluate effectiveness and safety of an antimicrobial stewardship intervention (comprising a training webinar, monthly feedback of antibiotic prescribing data from EHRs, and electronic decision support tools)
Overall, the intervention was associated with moderately reduced antibiotic prescribing, with no evidence of increased serious bacterial complications; the intervention reduced prescribing for adults but not for children or people aged 85 years or older
Multifaceted interventions, drawing on EHR data, could be scaled up to promote effective antimicrobial stewardship in primary care; the needs of very young or old patients require further consideration

## Web extra

Extra material supplied by authors

**Web appendix:** Supplementary data

## Notes

Contributors: MCG contributed to the trial design, conduct, and analysis; ATP contributed to the trial design, statistical analysis plan, and statistical advice; JC contributed to the intervention delivery and trial analysis; DJ, LM, and LY contributed to the design of the interventions and evaluation; JS, KS, and MW contributed to the recruitment and coordination of trial; and RF, ADH, PL, MVM, and MA contributed to the design of the study, intervention development, and interpretation of results. All authors contributed to and approved the final manuscript. The authors had full access to all the data in the study and all authors shared final responsibility for the decision to submit for publication. MG is guarantor. The corresponding author attests that all listed authors meet authorship criteria and that no others meeting the criteria have been omitted.

Trial steering committee: Jackie Cassell (chair), Susan Hopkins (Public Health England), Tim Chadborn (Public Health England), Nanik Pursani (lay member). Data monitoring committee: Christine A’Court (chair), Helen Strongman (CPRD/MHRA), Derek Cook (St George’s, University of London), Jason Oke (University of Oxford).

Data sharing: Requests for access to data from the study should be addressed to the corresponding author at martin.gulliford@kcl.ac.uk. The study protocol has been published. All proposals requesting data access will need to specify how it is planned to use the data, and all proposals will need approval of the trial co-investigator team, and CPRD before data release.

The lead author affirms that the manuscript is an honest, accurate, and transparent account of the study being reported; that no important aspects of the study have been omitted; and that any discrepancies from the study as planned (and, if relevant, registered) have been explained.
