# Supplementary material for: Effectiveness and safety of electronically delivered prescribing feedback and decision support on antibiotic use for respiratory illness in primary care: REDUCE cluster randomised trial
Source: BMJ. 2019 Feb 13;364:l236. doi: 10.1136/bmj.l236 (PMC6371944; doi:10.1136/bmj.l236)
Supplement: Supplementary file 1 — Web appendix: Supplementary data [file gulm047010.ww.pdf]

REDUCE Trial: Supplementary Data.

Supplementary Table 1: Results of sensitivity analysis comparing analysis with different time-windows for excluding secondary consultations.

Measure	AMS intervention (323,155·4 person-years)	Usual care (259,519·7 person-years)	Adjusted RR (95% confidence interval)[a]	P Value
**Base case: 14-day time window**				
RTI consultations	78,324	66,114		
AB prescriptions	31,907	27,923		
AB Prescription rate (per 1000 person-years)	98.7	107.6	0.88 (0.78 to 0.99)	0.04
**10-day time window**				
RTI consultations	80,160	67,695		
AB prescriptions	32,643	28,567		
AB Prescription rate (per 1000 person-years)	101.0	110.1	0.88 (0.78 to 0.99)	0.04
**No time window**				
RTI consultations	86,473	72,717		
AB prescriptions	35,271	30,549		
AB Prescription rate (per 1000 person-years)	109.1	117.7	0.88 (0.77 to 0.99)	0.04

AB, antibiotic; AMS, antimicrobial stewardship; RR, rate ratio; RTI, self-limiting respiratory tract infection
[a]adjusted for random effect of general practice and fixed effects of gender, age-group, comorbidity, region, quarter in study, practice-specific baseline rate and interaction with period of randomisation

Supplementary Table 2: Effect of intervention on secondary outcome measures. Figures are frequencies except where indicated.

	Measure	Antimicrobial stewardship intervention	Usual care	RR (95% confidence interval)[a]	P Value
**RTI Consultation Rate**	RTI consultations	78,324	66,114	0.94 (0·86 to 1·03)	0·19
	Person-years	323,155·4	259,519·7		
	Crude rate (per, 1000)	242·4	254·8		
**Proportion of RTI consultations with AB prescribed**	AB prescriptions	31,907	27,923	0·96 (0·89 to 1·03)	0·25
	RTI consultations	78,324	66,114		
	Proportion (%)	40·7	42·2		
**Rate of AB Prescribing for all indications**	AB prescriptions	185,924	150,539	0·93 (0·83 to 1·04)	0·18
	Person-years	323,155·4	259,519·7		
	Crude rate (per, 1000)	575·3	581·0		

AB, antibiotic; RR, rate ratio; RTI, self-limiting respiratory tract infection
[a]adjusted for random effect of general practice and fixed effects of gender, age-group, comorbidity, region, quarter in study, practice-specific baseline rate and interaction with period of randomisation

Supplementary Table 3: Effect of intervention on primary outcome for children, adults and people aged 85 and older separately.

	**AMS intervention AB / Person-time**	**Usual care AB / Person-time**	**RR (95%CI)[a]**
Children 0:14 years	7,497 / 53,826·3	6,432 / 46,019·6	0·96 (0·82 to 1·12)
Adults 15:84 years	23,551 / 261,841·3	20,811 / 207,611·4	0·84 (0·75 to 0·95)
Adults 85+ years	859 / 7,487·8	680 / 5,888·7	0·97 (0·79 to 1·18)

AB, antibiotic prescriptions; AMS, antimicrobial stewardship; RR, rate ratio; RTI, self-limiting respiratory tract infection consultations
[a]adjusted for random effect of general practice and fixed effects of gender, age-group, comorbidity, region, quarter in study, practice-specific baseline rate and interaction with period

Supplementary Table 4: Effect of intervention by sub-groups of practice-level covariates. Figures are frequencies except where indicated.

Practice-level covariate		AMS intervention AB / Person-time	Usual care AB / Person-time	RR (95%CI)[a]	P Interaction[b]
Region	South and East	5,460 / 67,062·7	6,674 / 61,281·7	0·73 (0·61 to 0·87)	0·03
	London	2,693 / 36,421·0	1,316 / 24,176·8	1·46 (0·53 to 4·06)	
	South West	3,225 / 39,329·9	2,158 / 23,026·7	0·81 (0·65 to 1·02)	
	North and Midlands	1,752 / 17,067·6	3,341 / 32,210·1	1·23 (1·03 to 1·48)	
	Wales	4,172 / 52,810·3	4,999 / 50,643·0	0·86 (0·70 to 1·06)	
	Scotland	8,396 / 76,041·1	5,076 / 42,807·1	0·71 (0·53 to 0·95)	
	Northern Ireland	6,209 / 34,422·9	4,359 / 25,374·3	1·00 (0·87 to 1·14)	
AB Prescribing Quartile	Lowest	5,516 / 109,110·8	1,882 / 39,425·9	0·87 (0·62 to 1·22)	0·48
	2	6,139 / 74,090·7	6,017 / 71,910·6	0·75 (0·55 to 1·01)	
	3	7,051 / 62,127·4	9,381 / 78,259·7	0·92 (0·75 to 1·13)	
	Highest	13,201 / 77,826·6	10,643,69,923·5	0·97 (0·86 to 1·10)	

AB, antibiotic prescriptions; AMS, antimicrobial stewardship; RR, rate ratio; RTI, self-limiting respiratory tract infection consultations

[a]adjusted for random effect of general practice and fixed effects of gender, age-group, comorbidity, region, quarter in study, practice-specific baseline rate and interaction with period

[b]test for interaction of trial arm with covariate

Supplementary Table 5: Antibiotic (AB) prescribing by type of RTI. (Cons., consultations; Pres., prescriptions).

Type of RTI	Intervention		Control		RR (95% CI)[a]
	Cons.	AB pres.	Cons.	AB pres.	
Colds and URTI	15,571	3,304	12,892	3,072	1·00 (0·69 to 1·44)
Cough and bronchitis	38,337	15,152	32,743	13,109	0·85 (0·71 to 1·03)
Otitis media	5,932	3,282	4,486	2,647	0·93 (0·75 to 1·14)
Rhinosinusitis	3,214	2,552	2,921	2,391	0·90 (0·69 to 1·18)
Sore throat	15,270	7,617	13,072	6,704	0·92 (0·79 to 1·08)

AB, antibiotic prescriptions for RTI; RR, rate ratio
[a]adjusted for random effect of general practice and fixed effects of gender, age-group, comorbidity, region, quarter in study, practice-specific baseline rate and interaction with period

Supplementary Figure 1: example of antibiotic prescribing report.

Reduce Trial

Practice Prescribing Report: February 2017

This is the ninth in a series of reports on your practice that you will receive during the REDUCE Trial. Collecting all 13 reports will provide evidence of audit suitable for use in appraisal.

Information about antibiotic prescribing at your Practice:

During the first six months of the Trial in your practice:

- There were 1714 consultations for RTI compared with 1882 in the same period last year
- The proportion of consultations with antibiotics prescribed was 57% compared with 58% in the same period last year
- There has been a a 1% reduction in antibiotic prescribing since the same time last year

	May 2015	Jun 2015	Jul 2015	Aug 2015	Sep 2015	Oct 2015	Nov 2015	Dec 2015	Jan 2016	Feb 2016	Mar 2016	Apr 2016	Total
RTI Consultations	176	210	163	160	230	259	337	347	314	276	304	245	3021
Antibiotic prescribed	99	107	103	93	139	154	172	229	170	181	184	147	1778
Percentage prescribed AB	56	51	63	58	60	59	51	66	54	66	61	60	59
	**May 2016**	**Jun 2016**	**Jul 2016**	**Aug 2016**	**Sep 2016**	**Oct 2016**	**Nov 2016**	**Dec 2016**	**Jan 2017**	**Feb 2017**	**Mar 2017**	**Apr 2017**	**Total**
RTI Consultations	232	207	149	184	206	220	258	258					1714
Antibiotic prescribed	147	123	97	112	111	110	123	160					983
Percentage prescribed AB	63	59	65	61	54	50	48	62					57
Change since last year (%)	7	8	2	3	-6	-9	-3	-4					-1

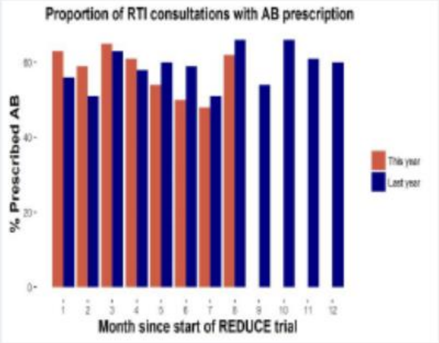

How to plan changes in antibiotic prescribing for your Practice

Patients can also be reassured that clinical trials and other research studies have shown that antibiotic prescription has little or no effect on the duration of illness or the severity of symptoms.

To help support patients, you can access the **Reduce Trial Support Tools** via DXS to print individualised information for patients.

For more information about this prescribing report please contact Professor Martin Gulliford at King's College London **martin.gulliford@kcl.ac.uk.**

Supplementary Figure 2: Map of decision support tools.

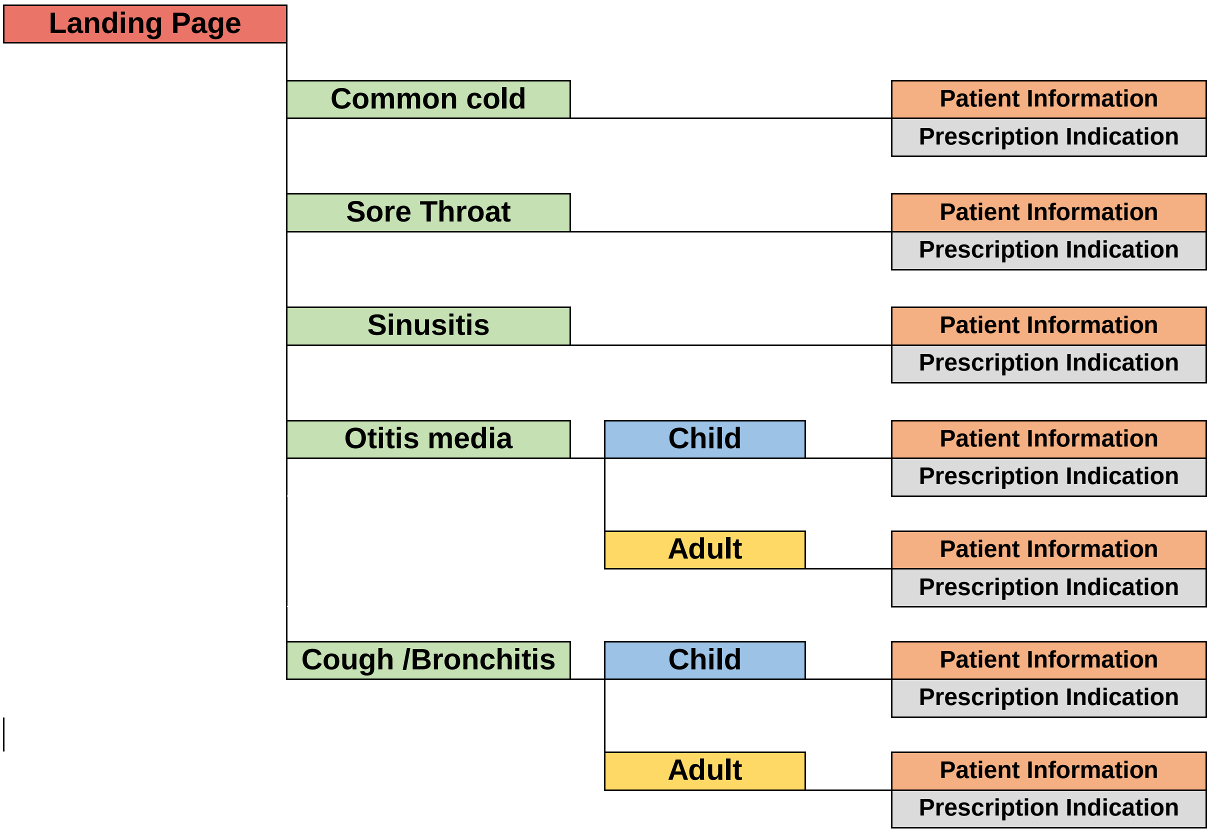

Supplementary Figure 3: Part of one of the REDUCE Trial patient information leaflets.

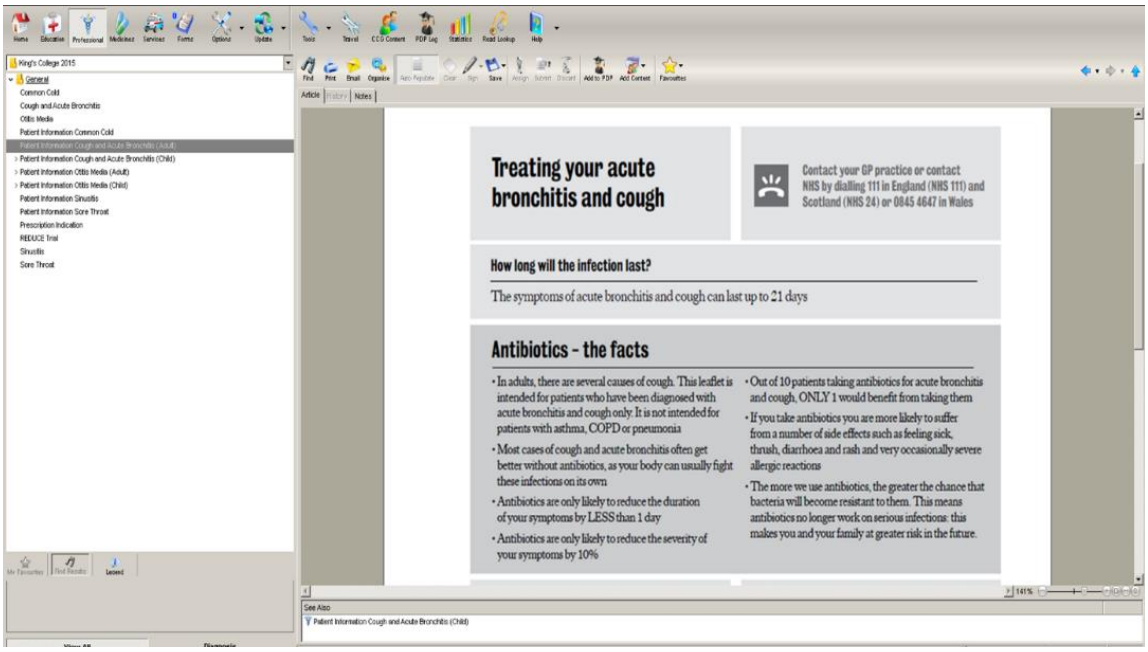

Supplementary Figure 4: Screenshot showing the REDUCE Trial prescription indication page.

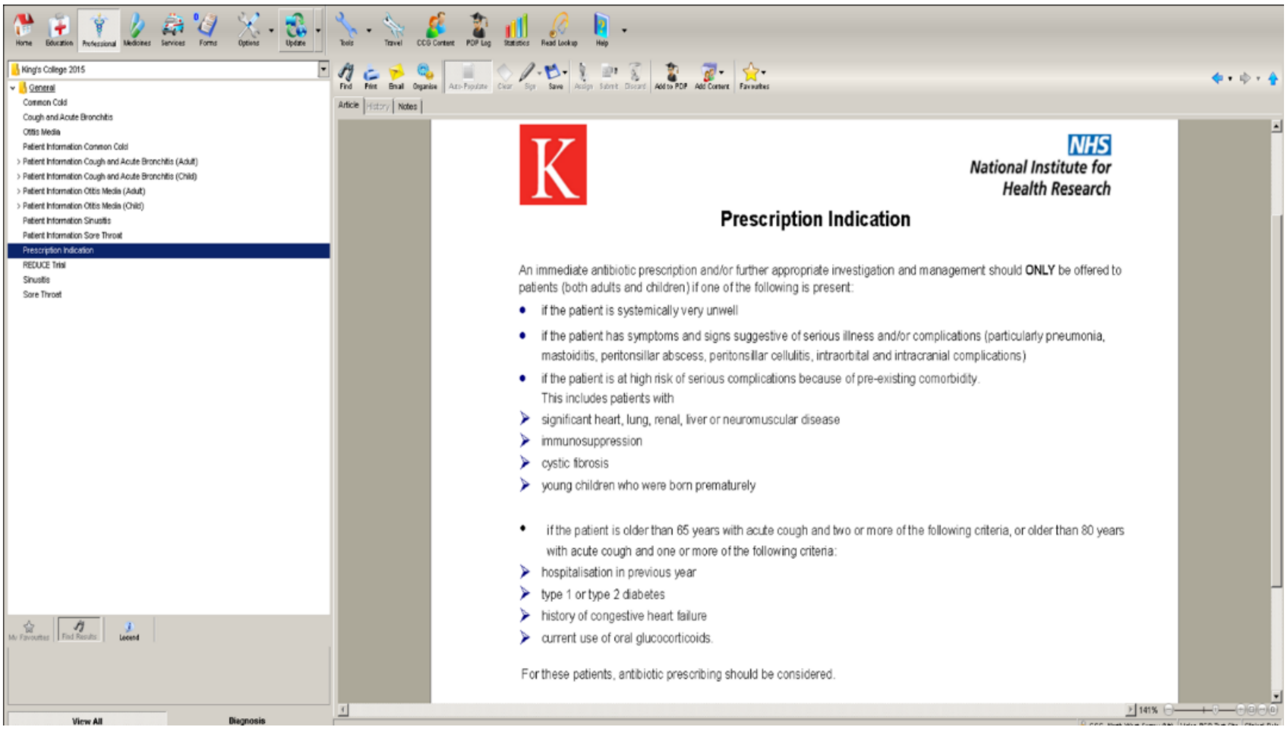

Supplementary table 6: RTI Read codes

readcode	readterm	class
1656	Feverish cold	colds, influenza and URTI
16L..00	Influenza-like symptoms	colds, influenza and URTI
H0...00	Acute respiratory infections	colds, influenza and URTI
H00..00	Acute nasopharyngitis	colds, influenza and URTI
H00..11	Common cold	colds, influenza and URTI
H00..12	Coryza - acute	colds, influenza and URTI
H00..13	Febrile cold	colds, influenza and URTI
H00..14	Nasal catarrh - acute	colds, influenza and URTI
H00..15	Pyrexial cold	colds, influenza and URTI
H00..16	Rhinitis - acute	colds, influenza and URTI
H05..00	Other acute upper respiratory infections	colds, influenza and URTI
H051.00	Acute upper respiratory tract infection	colds, influenza and URTI
H054.00	Recurrent upper respiratory tract infection	colds, influenza and URTI
H05y.00	Other upper respiratory infections of multiple sites	colds, influenza and URTI
H05z.00	Upper respiratory infection NOS	colds, influenza and URTI
H05z.11	Upper respiratory tract infection NOS	colds, influenza and URTI
H05z.12	Viral upper respiratory tract infection NOS	colds, influenza and URTI
H06z111	Respiratory tract infection	colds, influenza and URTI
H07..00	Chest cold	colds, influenza and URTI
H0y..00	Other specified acute respiratory infections	colds, influenza and URTI
H0z..00	Acute respiratory infection NOS	colds, influenza and URTI
H27..00	Influenza	colds, influenza and URTI
H271.00	Influenza with other respiratory manifestation	colds, influenza and URTI
H271z00	Influenza with respiratory manifestations NOS	colds, influenza and URTI
H27y.00	Influenza with other manifestations	colds, influenza and URTI
H27y100	Influenza with gastrointestinal tract involvement	colds, influenza and URTI
H27yz00	Influenza with other manifestations NOS	colds, influenza and URTI
H27z.00	Influenza NOS	colds, influenza and URTI
H27z.11	Flu like illness	colds, influenza and URTI
H27z.12	Influenza like illness	colds, influenza and URTI
H29..00	Avian influenza	colds, influenza and URTI
H2A..00	Influenza due to Influenza A virus subtype H1N1	colds, influenza and URTI
H2A..11	Influenza A (H1N1) swine flu	colds, influenza and URTI
Hyu0.00	[X]Acute upper respiratory infections	colds, influenza and URTI
Hyu0300	[X]Other acute upper respiratory infections/multiple sites	colds, influenza and URTI
Hyu0500	[X]Influenza+other manifestations,influenza virus	colds, influenza and URTI
Hyu0600	[X]Influenza+oth respiratory manifestatns,virus not	colds, influenza and URTI
Hyu0700	[X]Influenza+other manifestations, virus not	colds, influenza and URTI
14B3.11	H/O: bronchitis	cough and bronchitis
171..00	Cough	cough and bronchitis
171..11	C/O - cough	cough and bronchitis
1712	Dry cough	cough and bronchitis

1713	Productive cough -clear sputum	cough and bronchitis
1714	Productive cough -green sputum	cough and bronchitis
1715	Productive cough-yellow sputum	cough and bronchitis
1716	Productive cough NOS	cough and bronchitis
1716.11	Coughing up phlegm	cough and bronchitis
1717	Night cough present	cough and bronchitis
1719	Chesty cough	cough and bronchitis
1719.11	Bronchial cough	cough and bronchitis
171A.00	Chronic cough	cough and bronchitis
171B.00	Persistent cough	cough and bronchitis
171C.00	Morning cough	cough and bronchitis
171D.00	Evening cough	cough and bronchitis
171E.00	Unexplained cough	cough and bronchitis
171F.00	Cough with fever	cough and bronchitis
171G.00	Bovine cough	cough and bronchitis
171H.00	Difficulty in coughing up sputum	cough and bronchitis
171J.00	Reflux cough	cough and bronchitis
171K.00	Barking cough	cough and bronchitis
171Z.00	Cough symptom NOS	cough and bronchitis
173B.00	Nocturnal cough / wheeze	cough and bronchitis
H06..00	Acute bronchitis and bronchiolitis	cough and bronchitis
H060.00	Acute bronchitis	cough and bronchitis
H060.11	Acute wheezy bronchitis	cough and bronchitis
H060000	Acute fibrinous bronchitis	cough and bronchitis
H060100	Acute membranous bronchitis	cough and bronchitis
H060200	Acute pseudomembranous bronchitis	cough and bronchitis
H060300	Acute purulent bronchitis	cough and bronchitis
H060400	Acute croupous bronchitis	cough and bronchitis
H060500	Acute tracheobronchitis	cough and bronchitis
H060600	Acute pneumococcal bronchitis	cough and bronchitis
H060700	Acute streptococcal bronchitis	cough and bronchitis
H060800	Acute haemophilus influenzae bronchitis	cough and bronchitis
H060900	Acute neisseria catarrhalis bronchitis	cough and bronchitis
H060A00	Acute bronchitis due to mycoplasma pneumoniae	cough and bronchitis
H060B00	Acute bronchitis due to coxsackievirus	cough and bronchitis
H060C00	Acute bronchitis due to parainfluenza virus	cough and bronchitis
H060D00	Acute bronchitis due to respiratory syncytial virus	cough and bronchitis
H060E00	Acute bronchitis due to rhinovirus	cough and bronchitis
H060F00	Acute bronchitis due to echovirus	cough and bronchitis
H060v00	Subacute bronchitis unspecified	cough and bronchitis
H060w00	Acute viral bronchitis unspecified	cough and bronchitis
H060x00	Acute bacterial bronchitis unspecified	cough and bronchitis
H060z00	Acute bronchitis NOS	cough and bronchitis
H061.00	Acute bronchiolitis	cough and bronchitis
H061000	Acute capillary bronchiolitis	cough and bronchitis
H061100	Acute obliterating bronchiolitis	cough and bronchitis

H061200	Acute bronchiolitis with bronchospasm	cough and bronchitis
H061300	Acute exudative bronchiolitis	cough and bronchitis
H061500	Acute bronchiolitis due to respiratory syncytial virus	cough and bronchitis
H061600	Acute bronchiolitis due to other specified organisms	cough and bronchitis
H061z00	Acute bronchiolitis NOS	cough and bronchitis
H06z.00	Acute bronchitis or bronchiolitis NOS	cough and bronchitis
H30..00	Bronchitis unspecified	cough and bronchitis
H30..11	Chest infection - unspecified bronchitis	cough and bronchitis
H300.00	Tracheobronchitis NOS	cough and bronchitis
H30z.00	Bronchitis NOS	cough and bronchitis
H310100	Smokers' cough	cough and bronchitis
Hyu1000	[X]Acute bronchitis due to other specified organisms	cough and bronchitis
Hyu1100	[X]Acute bronchiolitis, other specified organisms	cough and bronchitis
R062.00	[D]Cough	cough and bronchitis
1C3..00	Earache symptoms	otitis-media
1C32.00	Unilateral earache	otitis-media
1C33.00	Bilateral earache	otitis-media
1C3Z.00	Earache symptom NOS	otitis-media
1c3..00		otitis-media
A552.00	Postmeasles otitis media	otitis-media
F51..00	Nonsuppurative otitis media + eustachian tube disorders	otitis-media
F510.00	Acute non suppurative otitis media	otitis-media
F510000	Acute otitis media with effusion	otitis-media
F510011	Acute secretory otitis media	otitis-media
F510100	Acute serous otitis media	otitis-media
F510200	Acute mucoid otitis media	otitis-media
F510300	Acute sanguinous otitis media	otitis-media
F510z00	Acute nonsuppurative otitis media NOS	otitis-media
F514.00	Unspecified nonsuppurative otitis media	otitis-media
F514100	Serous otitis media NOS	otitis-media
F514200	Catarrhal otitis media NOS	otitis-media
F514300	Mucoid otitis media NOS	otitis-media
F514z00	Nonsuppurative otitis media NOS	otitis-media
F52..00	Suppurative and unspecified otitis media	otitis-media
F520.00	Acute suppurative otitis media	otitis-media
F520000	Acute suppurative otitis media tympanic membrane intact	otitis-media
F520100	Acute otitis media tympanic membrane ruptured	otitis-media
F520300	Acute suppurative otitis media due to disease EC	otitis-media
F520z00	Acute suppurative otitis media NOS	otitis-media
F524.00	Purulent otitis media NOS	otitis-media
F524000	Bilateral suppurative otitis media	otitis-media
F525.00	Recurrent acute otitis media	otitis-media
F526.00	Acute left otitis media	otitis-media
F527.00	Acute right otitis media	otitis-media

F528.00	Acute bilateral otitis media	otitis-media
F52z.00	Otitis media NOS	otitis-media
F52z.11	Infection ear	otitis-media
F540.00	Acute myringitis without otitis media	otitis-media
F540z00	Acute myringitis NOS	otitis-media
FyuP000	[X]Other acute nonsuppurative otitis media	otitis-media
FyuP200	[X]Other chronic suppurative otitis media	otitis-media
FyuP300	[X]Otitis media in bacterial diseases	otitis-media
FyuP400	[X]Otitis media in viral diseases classified elsewhere	otitis-media
FyuP500	[X]Otitis media in other diseases classified	otitis-media
SN30.11	Aero-otitis media	otitis-media
H01..00	Acute sinusitis	rhinosinusitis
H01..11	Sinusitis	rhinosinusitis
H010.00	Acute maxillary sinusitis	rhinosinusitis
H010.11	Antritis - acute	rhinosinusitis
H011.00	Acute frontal sinusitis	rhinosinusitis
H012.00	Acute ethmoidal sinusitis	rhinosinusitis
H013.00	Acute sphenoidal sinusitis	rhinosinusitis
H014.00	Acute rhinosinusitis	rhinosinusitis
H01y.00	Other acute sinusitis	rhinosinusitis
H01y000	Acute pansinusitis	rhinosinusitis
H01yz00	Other acute sinusitis NOS	rhinosinusitis
H01z.00	Acute sinusitis NOS	rhinosinusitis
H130.12	Maxillary sinusitis	rhinosinusitis
H131.11	Frontal sinusitis	rhinosinusitis
H135.00	Recurrent sinusitis	rhinosinusitis
H13y100	Pansinusitis	rhinosinusitis
Hyu0000	[X]Other acute sinusitis	rhinosinusitis
SN31.11	Aerosinusitis	rhinosinusitis
1C9..00	Sore throat symptom	sorethroat
1C9..11	Throat soreness	sorethroat
1C92.00	Has a sore throat	sorethroat
1C93.00	Persistent sore throat	sorethroat
1C9Z.00	Sore throat symptom NOS	sorethroat
1CB3.00	Throat pain	sorethroat
1CB3.11	Pain in throat	sorethroat
2DB6.00	O/E - follicular tonsillitis	sorethroat
2DC2.00	O/E - granular pharyngitis	sorethroat
2DC3.00	Inflamed throat	sorethroat
A34..00	Streptococcal sore throat and scarlatina	sorethroat
A340.00	Streptococcal sore throat	sorethroat
A340100	Streptococcal laryngitis	sorethroat
A340200	Streptococcal pharyngitis	sorethroat
A340300	Streptococcal tonsillitis	sorethroat
A340z00	Streptococcal sore throat NOS	sorethroat
A34z.00	Streptococcal sore throat with scarlatina NOS	sorethroat

A383000	Fusobacterial necrotising tonsillitis	sorethroat
AA12.00	Vincent's pharyngitis	sorethroat
AA1z.11	Vincent's laryngitis	sorethroat
AA1z.12	Vincent's tonsillitis	sorethroat
AA25.11	Rhinopharyngitis mutilans	sorethroat
H02..00	Acute pharyngitis	sorethroat
H02..11	Sore throat NOS	sorethroat
H02..12	Viral sore throat NOS	sorethroat
H02..13	Throat infection - pharyngitis	sorethroat
H020.00	Acute gangrenous pharyngitis	sorethroat
H021.00	Acute phlegmonous pharyngitis	sorethroat
H022.00	Acute ulcerative pharyngitis	sorethroat
H023.00	Acute bacterial pharyngitis	sorethroat
H023000	Acute pneumococcal pharyngitis	sorethroat
H023100	Acute staphylococcal pharyngitis	sorethroat
H023z00	Acute bacterial pharyngitis NOS	sorethroat
H024.00	Acute viral pharyngitis	sorethroat
H02z.00	Acute pharyngitis NOS	sorethroat
H03..00	Acute tonsillitis	sorethroat
H03..11	Throat infection - tonsillitis	sorethroat
H03..12	Tonsillitis	sorethroat
H030.00	Acute erythematous tonsillitis	sorethroat
H031.00	Acute follicular tonsillitis	sorethroat
H032.00	Acute ulcerative tonsillitis	sorethroat
H033.00	Acute catarrhal tonsillitis	sorethroat
H034.00	Acute gangrenous tonsillitis	sorethroat
H035.00	Acute bacterial tonsillitis	sorethroat
H035000	Acute pneumococcal tonsillitis	sorethroat
H035100	Acute staphylococcal tonsillitis	sorethroat
H035z00	Acute bacterial tonsillitis NOS	sorethroat
H036.00	Acute viral tonsillitis	sorethroat
H037.00	Recurrent acute tonsillitis	sorethroat
H03z.00	Acute tonsillitis NOS	sorethroat
H04..00	Acute laryngitis and tracheitis	sorethroat
H040.00	Acute laryngitis	sorethroat
H040000	Acute oedematous laryngitis	sorethroat
H040100	Acute ulcerative laryngitis	sorethroat
H040200	Acute catarrhal laryngitis	sorethroat
H040300	Acute phlegmonous laryngitis	sorethroat
H040400	Acute haemophilus influenzae laryngitis	sorethroat
H040600	Acute suppurative laryngitis	sorethroat
H040w00	Acute viral laryngitis unspecified	sorethroat
H040x00	Acute bacterial laryngitis unspecified	sorethroat
H040z00	Acute laryngitis NOS	sorethroat
H041.00	Acute tracheitis	sorethroat
H041000	Acute tracheitis without obstruction	sorethroat

H041100	Acute tracheitis with obstruction	sorethroat
H041z00	Acute tracheitis NOS	sorethroat
H042.00	Acute laryngotracheitis	sorethroat
H042.11	Laryngotracheitis	sorethroat
H042000	Acute laryngotracheitis without obstruction	sorethroat
H042100	Acute laryngotracheitis with obstruction	sorethroat
H042z00	Acute laryngotracheitis NOS	sorethroat
H043.00	Acute epiglottitis (non strep)	sorethroat
H043.11	Viral epiglottitis	sorethroat
H043000	Acute epiglottitis without obstruction	sorethroat
H043100	Acute epiglottitis with obstruction	sorethroat
H043200	Acute obstructive laryngitis	sorethroat
H043211	Croup	sorethroat
H043z00	Acute epiglottitis NOS	sorethroat
H044.00	Croup	sorethroat
H04z.00	Acute laryngitis and tracheitis NOS	sorethroat
H050.00	Acute laryngopharyngitis	sorethroat
H052.00	Pharyngotracheitis	sorethroat
H053.00	Tracheopharyngitis	sorethroat
H055.00	Pharyngolaryngitis	sorethroat
H121100	Atrophic pharyngitis	sorethroat
H121200	Granular pharyngitis	sorethroat
H121300	Hypertrophic pharyngitis	sorethroat
H121400	Pharyngitis keratosa	sorethroat
H14y600	Lingular tonsillitis	sorethroat
H271000	Influenza with laryngitis	sorethroat
H271100	Influenza with pharyngitis	sorethroat
H301.00	Laryngotracheobronchitis	sorethroat
Hyu0100	[X]Acute pharyngitis due to other specified organisms	sorethroat
Hyu0200	[X]Acute tonsillitis due to other specified organisms	sorethroat
R041.00	[D]Throat pain	sorethroat

Supplementary table 7: Safety outcomes Read codes

readcode	readterm	class
H50..00	Empyema	Empyema
H501100	Thorax abscess NOS	Empyema
H50z.00	Empyema NOS	Empyema
H501200	Pleural empyema	Empyema
H501400	Purulent pleurisy	Empyema
H500100	Empyema with bronchopleural fistula	Empyema
H501000	Pleural abscess	Empyema
H501500	Pyopneumothorax	Empyema
H501300	Lung empyema NOS	Empyema
H500.00	Empyema with fistula	Empyema
H501600	Pyothorax	Empyema
H501.00	Empyema with no fistula	Empyema
H500400	Empyema with pleural fistula NOS	Empyema
H500000	Empyema with bronchocutaneous fistula	Empyema
F040011	Cerebral abscess	ICRA
F040.11	Brain abscess	ICRA
F040.00	Intracranial abscess	ICRA
F040111	Cerebellar abscess	ICRA
F040000	Cerebral intracranial abscess	ICRA
F040511	Subdural intracranial abscess	ICRA
F040400	Extradural intracranial abscess	ICRA
F040311	Epidural intracranial abscess	ICRA
F040100	Cerebellar intracranial abscess	ICRA
F040500	Subdural intracranial abscess	ICRA
F040211	Otogenic intracranial abscess	ICRA
F040z00	Intracranial abscess NOS	ICRA
F040200	Otogenic intracranial abscess	ICRA
F040300	Epidural intracranial abscess	ICRA
A383011	Lemierre's syndrome	Lemierre
F53z.00	Mastoiditis NOS	MAS
F53..00	Mastoiditis and related conditions	MAS
F530.00	Acute mastoiditis	MAS
F531.00	Chronic mastoiditis	MAS
F531z00	Chronic mastoiditis NOS	MAS
F530.11	Abscess of mastoid	MAS
F530z00	Acute mastoiditis NOS	MAS
F530000	Acute mastoiditis without complications	MAS
F530100	Subperiosteal mastoid abscess	MAS
F530300	Acute mastoiditis with other complication	MAS
FyuP700	[X]Other mastoiditis and related conditions	MAS
F530.12	Empyema of mastoid	MAS
A360.00	Meningococcal meningitis	Meningitis
F00..00	Bacterial meningitis	Meningitis

A362.00	Meningococcal septicaemia	Meningitis
F001.00	Pneumococcal meningitis	Meningitis
F000.00	Haemophilus meningitis	Meningitis
A36..00	Meningococcal infection	Meningitis
F005.00	Meningitis - meningococcal	Meningitis
A366.00	Meningococcal meningitis with meningococcal septicaemia	Meningitis
A365.00	Meningococcal meningitis with acute meningococcal septicaem	Meningitis
F002.00	Streptococcal meningitis	Meningitis
A361.00	Meningococcal encephalitis	Meningitis
F033011	Meningococcal encephalitis	Meningitis
F00z.00	Bacterial meningitis NOS	Meningitis
A36z.00	Meningococcal infection NOS	Meningitis
A362000	Acute meningococcaemia	Meningitis
F033000	Encephalitis due to meningococcus	Meningitis
F00y.00	Other specified bacterial meningitis	Meningitis
F00yz00	Other specified bacterial meningitis NOS	Meningitis
Fyu0000	[X]Other bacterial meningitis	Meningitis
H15..11	Quinsy	PTA
H15..00	Peritonsillar abscess - quinsy	PTA
7531100	Drainage of peritonsillar abscess	PTA
7531111	Drainage of quinsy	PTA
2DB5.11	O/E - quinsy present	PTA
2DB5.00	O/E - tonsils - quinsy present	PTA
A341.00	Scarlet fever - scarlatina	ScarletFever
A341.11	Scarlet fever	ScarletFever
A341.12	Scarlatina	ScarletFever
65V7.00	Notification of scarlet fever	ScarletFever
1414	H/O: scarlatina	ScarletFever
A34..00	Streptococcal sore throat and scarlatina	ScarletFever
A34z.00	Streptococcal sore throat with scarlatina NOS	ScarletFever
A57y400	Pseudoscarlatina	ScarletFever
A57y300	Parascarlatina	ScarletFever
N300.00	Acute osteomyelitis	osteomyelitis
N302.00	Unspecified osteomyelitis	osteomyelitis
N302000	Unspecified osteomyelitis of unspecified site	osteomyelitis
N300600	Acute osteomyelitis of the lower leg	osteomyelitis
N302500	Unspecified osteomyelitis of the pelvic region and thigh	osteomyelitis
N300511	Hip acute osteomyelitis	osteomyelitis
N300700	Acute osteomyelitis of the ankle and foot	osteomyelitis
N302z00	Unspecified osteomyelitis NOS	osteomyelitis
N300z00	Acute osteomyelitis NOS	osteomyelitis
J064.12	Osteomyelitis - jaw	osteomyelitis
N302700	Unspecified osteomyelitis of the ankle and foot	osteomyelitis
N302600	Unspecified osteomyelitis of the lower leg	osteomyelitis
N300Q00	Acute osteomyelitis-femur	osteomyelitis

N300500	Acute osteomyelitis of the pelvic region and thigh	osteomyelitis
N300000	Acute osteomyelitis of unspecified site	osteomyelitis
N300S00	Acute osteomyelitis-tibia	osteomyelitis
N300Y00	Acute osteomyelitis-phalanx of toe	osteomyelitis
N300800	Acute osteomyelitis of other specified site	osteomyelitis
N302800	Unspecified osteomyelitis of other specified site	osteomyelitis
N300100	Acute osteomyelitis of the shoulder region	osteomyelitis
N300Z00	Acute haematogenous osteomyelitis	osteomyelitis
N300400	Acute osteomyelitis of the hand	osteomyelitis
N300712	Foot - acute osteomyelitis	osteomyelitis
A022400	Salmonella osteomyelitis	osteomyelitis
N300X00	Acute osteomyelitis-metatarsal	osteomyelitis
N302400	Unspecified osteomyelitis of the hand	osteomyelitis
NyuC300	[X]Other osteomyelitis	osteomyelitis
N300C00	Acute osteomyelitis-lumbar spine	osteomyelitis
N300N00	Acute osteomyelitis-phalanx of finger/thumb	osteomyelitis
N300200	Acute osteomyelitis of the upper arm	osteomyelitis
F4G0400	Orbital osteomyelitis	osteomyelitis
N300711	Ankle - acute osteomyelitis	osteomyelitis
N302a00	Osteomyelitis of vertebra	osteomyelitis
N309.00	Subacute osteomyelitis	osteomyelitis
N300U00	Acute osteomyelitis-calcaneum	osteomyelitis
N302100	Unspecified osteomyelitis of the shoulder region	osteomyelitis
N300300	Acute osteomyelitis of the forearm	osteomyelitis
N300T00	Acute osteomyelitis-fibula	osteomyelitis
N302200	Unspecified osteomyelitis of the upper arm	osteomyelitis
N302300	Unspecified osteomyelitis of the forearm	osteomyelitis
N300H00	Acute osteomyelitis-humerus	osteomyelitis
N300J00	Acute osteomyelitis-radius	osteomyelitis
N300F00	Acute osteomyelitis-clavicle	osteomyelitis
N300A00	Acute osteomyelitis-cervical spine	osteomyelitis
N300B00	Acute osteomyelitis-thoracic spine	osteomyelitis
N300P00	Acute osteomyelitis-pelvis	osteomyelitis
N300512	Pelvis acute osteomyelitis	osteomyelitis
N300513	Thigh acute osteomyelitis	osteomyelitis
N300R00	Acute osteomyelitis-patella	osteomyelitis
N300V00	Acute osteomyelitis-talus	osteomyelitis
N300M00	Acute osteomyelitis-metacarpal	osteomyelitis
N300D00	Acute osteomyelitis-sacrum	osteomyelitis
N302900	Unspecified osteomyelitis of multiple sites	osteomyelitis
NyuC000	[X]Other acute osteomyelitis	osteomyelitis
N300K00	Acute osteomyelitis-ulna	osteomyelitis
J064400	Acute osteomyelitis of jaw	osteomyelitis
N300W00	Acute osteomyelitis-other tarsal bone	osteomyelitis
N300E00	Acute osteomyelitis-coccyx	osteomyelitis
N300900	Acute osteomyelitis of multiple sites	osteomyelitis

H26..00	Pneumonia due to unspecified organism	pneumonia
H25..00	Bronchopneumonia due to unspecified organism	pneumonia
H21..00	Lobar (pneumococcal) pneumonia	pneumonia
H260.00	Lobar pneumonia due to unspecified organism	pneumonia
H261.00	Basal pneumonia due to unspecified organism	pneumonia
H2B..00	Community acquired pneumonia	pneumonia
H28..00	Atypical pneumonia	pneumonia
H260000	Lung consolidation	pneumonia
H231.00	Pneumonia due to mycoplasma pneumoniae	pneumonia
H22z.00	Bacterial pneumonia NOS	pneumonia
H2C..00	Hospital acquired pneumonia	pneumonia
H22..00	Other bacterial pneumonia	pneumonia
H25..11	Chest infection - unspecified bronchopneumonia	pneumonia
H23..00	Pneumonia due to other specified organisms	pneumonia
H263.00	Pneumonitis, unspecified	pneumonia
H223.00	Pneumonia due to streptococcus	pneumonia
H22y200	Pneumonia - Legionella	pneumonia
H26..11	Chest infection - pnemonia due to unspecified organism	pneumonia
H22..11	Chest infection - other bacterial pneumonia	pneumonia
H224.00	Pneumonia due to staphylococcus	pneumonia
H23z.00	Pneumonia due to specified organism NOS	pneumonia
H22yz00	Pneumonia due to bacteria NOS	pneumonia
H220.00	Pneumonia due to klebsiella pneumoniae	pneumonia
H262.00	Postoperative pneumonia	pneumonia
H221.00	Pneumonia due to pseudomonas	pneumonia
H24y200	Pneumonia with pneumocystis carinii	pneumonia
H222.00	Pneumonia due to haemophilus influenzae	pneumonia
H233.00	Chlamydial pneumonia	pneumonia
H243.00	Pneumonia with whooping cough	pneumonia
H24..00	Pneumonia with infectious diseases EC	pneumonia
H243.11	Pneumonia with pertussis	pneumonia
H22y.00	Pneumonia due to other specified bacteria	pneumonia
H21..11	Chest infection - pneumococcal pneumonia	pneumonia
H23..11	Chest infection - pneumonia organism OS	pneumonia
H24..11	Chest infection with infectious disease EC	pneumonia
H246.00	Pneumonia with aspergillosis	pneumonia
H24y000	Pneumonia with actinomycosis	pneumonia
H223000	Pneumonia due to streptococcus, group B	pneumonia
H222.11	Pneumonia due to haemophilus influenzae	pneumonia
H22y000	Pneumonia due to escherichia coli	pneumonia
H24z.00	Pneumonia with infectious diseases EC NOS	pneumonia
H247000	Pneumonia with candidiasis	pneumonia
H24y.00	Pneumonia with other infectious diseases EC	pneumonia
H242.00	Pneumonia with ornithosis	pneumonia
H22y011	E.coli pneumonia	pneumonia
H24y400	Pneumonia with salmonellosis	pneumonia

H24y300	Pneumonia with Q-fever	pneumonia
H22yX00	Pneumonia due to other aerobic gram-negative bacteria	pneumonia
H24yz00	Pneumonia with other infectious diseases EC NOS	pneumonia
H24y600	Pneumonia with typhoid fever	pneumonia
H232.00	Pneumonia due to pleuropneumonia like organisms	pneumonia
H24y100	Pneumonia with nocardiasis	pneumonia
H230.00	Pneumonia due to Eaton's agent	pneumonia
H22y100	Pneumonia due to proteus	pneumonia
H247z00	Pneumonia with systemic mycosis NOS	pneumonia
H244.00	Pneumonia with tularaemia	pneumonia
H247100	Pneumonia with coccidioidomycosis	pneumonia
H24y500	Pneumonia with toxoplasmosis	pneumonia
K101.00	Acute pyelonephritis	pyelonephritis
K10y000	Pyelonephritis unspecified	pyelonephritis
K101z00	Acute pyelonephritis NOS	pyelonephritis
K10yz00	Unspecified pyelonephritis NOS	pyelonephritis
K10y.00	Pyelonephritis and pyonephrosis unspecified	pyelonephritis
K101000	Acute pyelonephritis without medullary necrosis	pyelonephritis
N010.11	Septic arthritis	SepticArthritis
N010.00	Pyogenic arthritis	SepticArthritis
N010y00	Pyogenic arthritis of other specified sites	SepticArthritis
N010600	Pyogenic arthritis of the lower leg	SepticArthritis
N010611	Knee pyogenic arthritis	SepticArthritis
N010800	Staphylococcal arthritis and polyarthritis	SepticArthritis
N010500	Pyogenic arthritis of the pelvic region and thigh	SepticArthritis
N010700	Pyogenic arthritis of the ankle and foot	SepticArthritis
N010z00	Pyogenic arthritis NOS	SepticArthritis
N010511	Hip pyogenic arthritis	SepticArthritis
N010100	Pyogenic arthritis of the shoulder region	SepticArthritis
N010400	Pyogenic arthritis of the hand	SepticArthritis
N010211	Elbow pyogenic arthritis	SepticArthritis
N010300	Pyogenic arthritis of the forearm	SepticArthritis
N010311	Wrist pyogenic arthritis	SepticArthritis
N010000	Pyogenic arthritis of unspecified site	SepticArthritis
N010200	Pyogenic arthritis of the upper arm	SepticArthritis
N010711	Ankle pyogenic arthritis	SepticArthritis
N010x00	Pyogenic arthritis of multiple sites	SepticArthritis
N010900	Pneumococcal arthritis and polyarthritis	SepticArthritis
A38z.00	Septicaemia NOS	TSSSepticaemia
A381.00	Staphylococcal septicaemia	TSSSepticaemia
A380.00	Streptococcal septicaemia	TSSSepticaemia
A382.00	Pneumococcal septicaemia	TSSSepticaemia
A384200	Escherichia coli septicaemia	TSSSepticaemia
A384211	E.coli septicaemia	TSSSepticaemia
A3Ay100	Toxic shock syndrome	TSSSepticaemia
A384.00	Septicaemia due to other gram negative organisms	TSSSepticaemia

A021.00	Salmonella septicaemia	TSSSepticaemia
A38y.00	Other specified septicaemias	TSSSepticaemia
A381000	Septicaemia due to Staphylococcus aureus	TSSSepticaemia
A380100	Septicaemia due to streptococcus, group B	TSSSepticaemia
A384300	Pseudomonas septicaemia	TSSSepticaemia
A384000	Gram negative septicaemia NOS	TSSSepticaemia
A384100	Haemophilus influenzae septicaemia	TSSSepticaemia
A380300	Septicaemia due to streptococcus pneumoniae	TSSSepticaemia
A380400	Septicaemia due to enterococcus	TSSSepticaemia
A380000	Septicaemia due to streptococcus, group A	TSSSepticaemia
L090z00	Septicaemia NOS following abortive pregnancy	TSSSepticaemia
A383.00	Septicaemia due to anaerobes	TSSSepticaemia
Ayu3J00	[X]Septicaemia, unspecified	TSSSepticaemia
A270100	Listeria septicaemia	TSSSepticaemia
A381100	Septicaemia due to coagulase-negative staphylococcus	TSSSepticaemia
R055200	[D]Endotoxic shock	TSSSepticaemia
A380500	Vancomycin resistant enterococcal septicaemia	TSSSepticaemia
Ayu3F00	[X]Streptococcal septicaemia, unspecified	TSSSepticaemia
A384z00	Other gram negative septicaemia NOS	TSSSepticaemia
Ayu3G00	[X]Septicaemia due to other gram-negative organisms	TSSSepticaemia
A271100	Erysipelothrix septicaemia	TSSSepticaemia
Ayu3E00	[X]Other streptococcal septicaemia	TSSSepticaemia
Ayu3H00	[X]Other specified septicaemia	TSSSepticaemia
